# Promotion of Ovarian Follicle Growth following mTOR Activation: Synergistic Effects of AKT Stimulators

**DOI:** 10.1371/journal.pone.0117769

**Published:** 2015-02-24

**Authors:** Yuan Cheng, Jaehong Kim, Xiao Xiao Li, Aaron J. Hsueh

**Affiliations:** Program of Reproductive and Stem Cell Biology, Department of Ob/Gyn, Stanford University School of Medicine, Stanford, CA, United States of America; University of Nevada School of Medicine, UNITED STATES

## Abstract

Mammalian target of rapamycin (mTOR) is a serine/threonine kinase and mTOR signaling is important in regulating cell growth and proliferation. Recent studies using oocyte- and granulosa cell-specific deletion of mTOR inhibitor genes TSC1 or TSC2 demonstrated the important role of mTOR signaling in the promotion of ovarian follicle development. We now report that treatment of ovaries from juvenile mice with an mTOR activator MHY1485 stimulated mTOR, S6K1 and rpS6 phosphorylation. Culturing ovaries for 4 days with MHY1485 increased ovarian explant weights and follicle development. In vivo studies further demonstrated that pre-incubation of these ovaries with MHY1485 for 2 days, followed by allo-grafting into kidney capsules of adult ovariectomized hosts for 5 days, led to marked increases in graft weights and promotion of follicle development. Mature oocytes derived from MHY1485-activated ovarian grafts could be successfully fertilized, leading the delivery of healthy pups. We further treated ovaries with the mTOR activator together with AKT activators (PTEN inhibitor and phosphoinositol-3-kinase stimulator) before grafting and found additive enhancement of follicle growth. Our studies demonstrate the ability of an mTOR activator in promoting follicle growth, leading to a potential strategy to stimulate preantral follicle growth in infertile patients.

## Introduction

Mammalian ovaries consist of follicles as basic functional units. During initial recruitment of follicles, unknown intraovarian mechanisms stimulate or release a small number of dormant primordial follicles to initiate growth [1]. Once entering the growing pool, ovarian follicles mature through primary, secondary, and antral stages to become preovulatory follicles containing mature oocytes [2]. Mammalian target of rapamycin (mTOR) is a serine/threonine kinase conserved from flies to mammals and part of the multi-protein mTORC1 complexes. Under the influence of nutritional factors, stress, oxygen, energy and other signals, the rapamycin-sensitive mTORC1 complex positively regulates cell growth and proliferation by promoting diverse anabolic processes, including biosynthesis of proteins, lipids and organelles, and by limiting catabolic processes such as autophagy [3,4]. In contrast, the tumor suppressor tuberous sclerosis complex 1 (TSC1) or 2 (TSC2) negatively regulates mTORC1 activity. Inactivating mutations of TSC1 or TSC2 result in tuberous sclerosis complex (TSC), a disease characterized by numerous benign tumors containing enlarged cells [5].

Studies using mutant mice indicated that oocyte-specific deletion of TSC1 or TSC2 promotes the growth of all primordial follicles in neonatal animals, leading to the exhaustion of the entire follicle pool, followed by a premature ovarian failure phenotype [6], [7]. Likewise, oocyte-specific deletion of the PTEN gene, upstream of AKT signaling, also increases AKT activity, followed by global activation of dormant ovarian follicles [8]. Of interest, double deletion of TSC1 and PTEN leads to synergistic enhancement of oocyte growth and follicle activation when compared with singly mutated mice [7], indicating that mTORC1 activation and increased AKT signaling synergistically stimulate the growth of primordial follicles. For larger follicles, mutant mice with disrupted TSC1 in granulosa cells of secondary follicles also exhibit enhanced follicle growth, leading to increased ovulatory capacity and delivery of more pups [9], followed by a premature ovarian failure phenotype [10]. Because AKT stimulation also promotes secondary follicle growth [11], it is becoming clear that mTOR and AKT signaling could act together to promote both primordial follicle activation and secondary follicle growth.

Taking advantage of the availability of an mTOR activator MHY1485 [12], we stimulated secondary follicle growth in juvenile mice using an in vitro activation-grafting approach [13] and derived preovulatory follicles containing mature oocytes.

## Results

### MHY1485 treatment stimulated phosphorylation of mTOR pathway proteins

**Fig 1:**
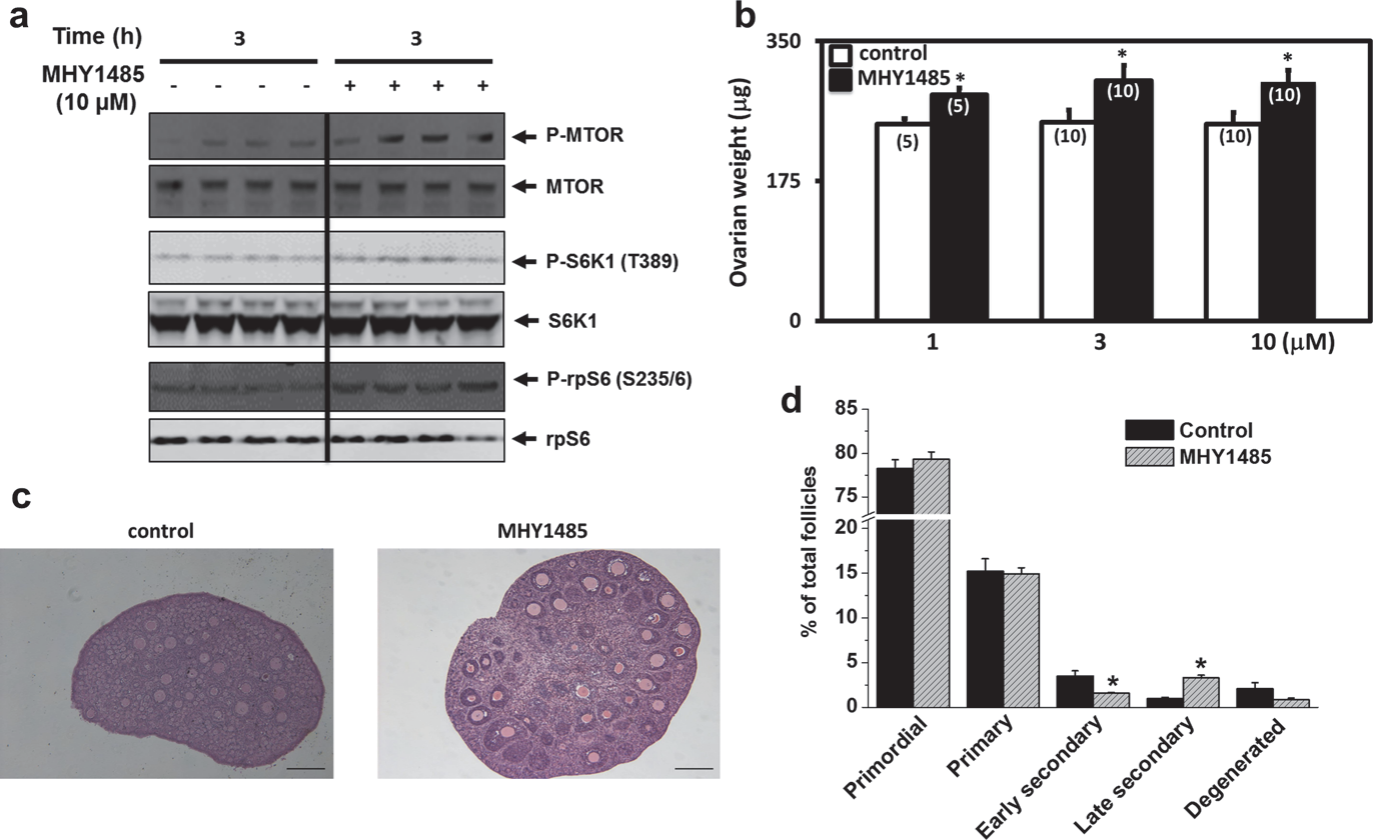
Treatment of ovaries with MHY1485 increased phosphorylation of mTOR pathway proteins and promoted secondary follicle development in vitro. A) Treatment of ovaries with MHY1485 increased phosphorylation of mTOR as well as S6K1 and rpS6. Ovaries from day 10 mice were treated with MHY1485 for 3h before immunoblotting. Blotting figures for each immunoblotting test were cropped from full-length blots that are presented in S1 Fig. B) Ovarian weight changes. Paired ovaries from day 10 mice were incubated with MHY1485 with media changes at day 2 of culture. At the end of 4 days of incubation, ovaries were fixed before weighing, followed by histological analyses. Numbers in parentheses denote number of ovaries used. * P<0.05. C) Ovarian histology; bars: 100 um. D) Follicle dynamics. N = 5. * P<0.05.

Based on recent studies showing the ability of MHY1485 to activate the mTOR pathway in rat hepatocyte and PC3 cell line [12,14], we investigated ovarian phosphorylation of mTOR and downstream proteins in this signaling pathway after MHY1485 treatment. Ovaries from day 10 mice were incubated for 3h with 10 uM of MHY1485 before immunoblotting analyses. As shown in Fig. 1A, treatment with MHY1485 increased phospho-mTOR levels without affecting total mTOR content. Activated mTORC1 complex phosphorylates Thr389 in ribosomal S6 kinase (S6K), thereby activating it to subsequently phosphorylate ribosomal protein S6 (rpS6) and promote ribosome biogenesis [15]. We further monitored S6K1 and rpS6 phosphorylation in ovarian tissues. As shown in Fig. 1A, MHY1485 treatment also increased the phosphorylation of downstream S6K1 and rpS6 proteins without affecting total S6K1 and rpS6 levels. These findings demonstrate the ability of MHY1485 to stimulate the mTOR signaling pathway in the ovary.

### Treatment with MHY1485 promoted follicle growth in vitro and in vivo

We treated ovaries from day 10 mice with increasing doses of MHY1485 using an explant culture model. As shown in Fig. 1B, treatment of ovaries for 4 days led to dose-dependent increases in ovarian weights. Histological analyses (Fig. 1C) and counting of follicles (Fig. 1D) indicated enhancement of follicle growth from early secondary to the late secondary stage.

**Fig 2:**
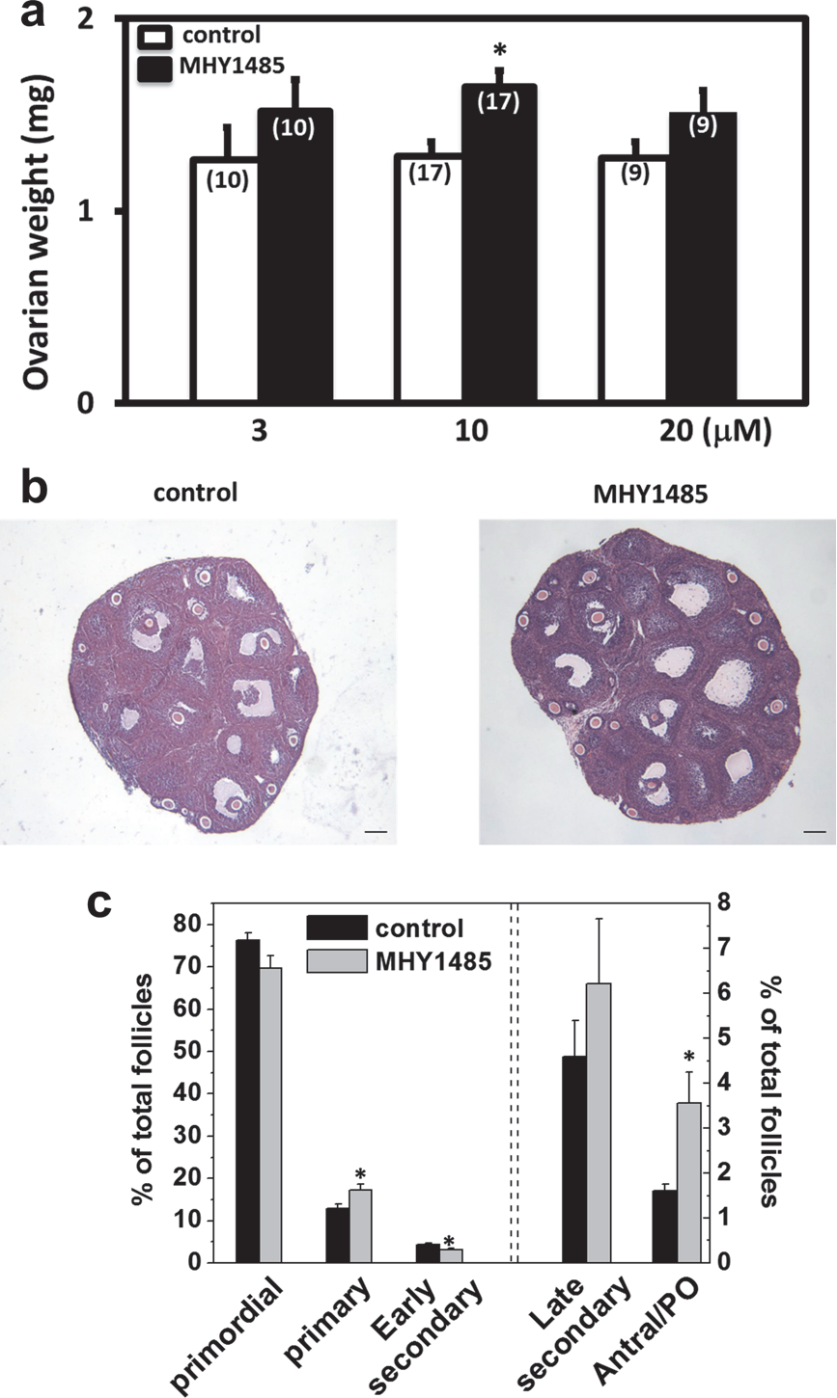
Short-term treatment of ovaries with MHY1485 followed by allo-transplantation promoted secondary follicle growth to the antral stage in ovarian grafts. A) Graft weight changes. Ovaries from day 10 mice were incubated with MHY1485 for 2 days, before grafting into adult ovariectomized hosts treated daily with FSH for 5 days. At the end of grafting, graft weights were determined and histological analyses were performed. Numbers in parentheses indicate number of grafts used. * P<0.05. B) Ovarian histology; bars: 100um. C) Follicle dynamics. PO: preovulatory. N = 5. * P<0.05.

**Fig 3:**
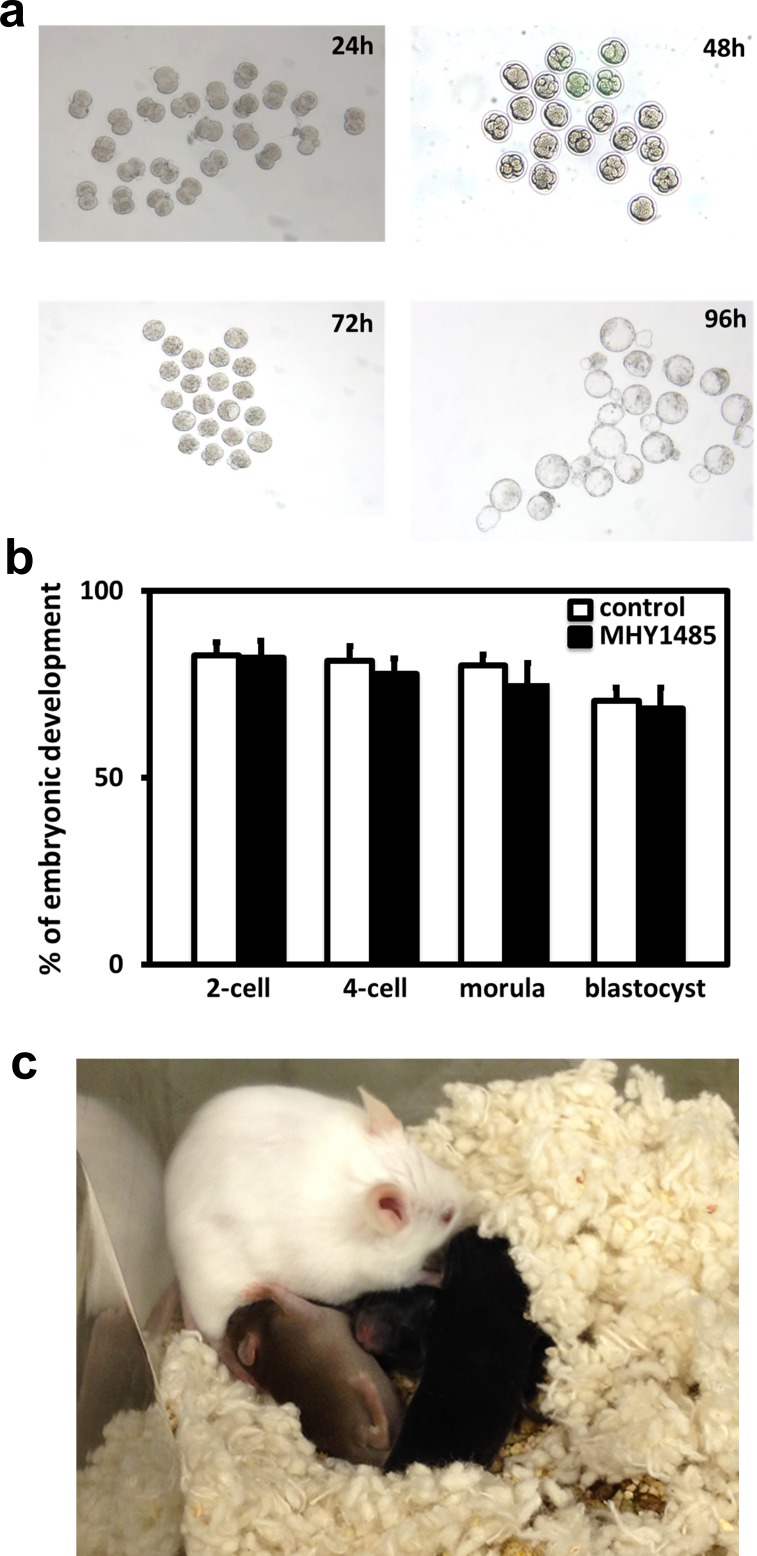
Treatment with MHY1485 and subsequent grafting allowed the derivation of mature oocytes and healthy offspring. A) Early embryonic development of oocytes after mTOR activator treatment. Ovaries were treated with MHY1485 for 2 days to activate follicles, followed by grafting into hosts for 5 days. Hosts were then treated with eCG and hCG. At 12h after hCG injection, mature oocytes were obtained and fertilized with sperm before culturing for 4 days. B) Percentage of oocytes developed into each embryonic stage. Early embryonic development for mice at 25 days of age served as controls. N = 30. C) Some 2-cell stage embryos were transferred into pseudopregnant hosts and pups were delivered.

We further treated day 10 ovaries with MHY1485 for 2 days in vitro, followed by grafting them into adult hosts treated daily with FSH for 5 days. As shown in Fig. 2A, treatment with MHY1485 increased graft weights. Following histological analyses (Fig. 2B) and follicle counting (Fig. 2C), increases in the development of antral/preovulatory follicles were apparent, together with a decrease of early secondary follicles and an increase of primary follicles. Using this model, we further treated the hosts with eCG for 2 days to promote the growth of preovulatory follicles, followed by an injection of hCG to promote oocyte maturation. At 12h after hCG injection, mature oocytes were punctured from preovulatory follicles for in vitro fertilization. As shown in Fig. 3A, oocytes obtained from MHY1485-pretreated ovaries could develop into blastocysts. As compared with mature oocytes obtained from day 25 mice without MHY1485 treatment, which served as controls, comparable early embryonic development was apparent based on the percentage of oocytes developing into each embryonic stage (Fig. 3B). Some of the 2-cell embryos derived from MHY1485-pretreated grafts were transferred into pseudopregnant surrogate mothers and healthy pups were delivered (Fig. 3C).

### Treatment with the mTOR activator augmented follicle growth promoted by AKT stimulators

**Fig 4:**
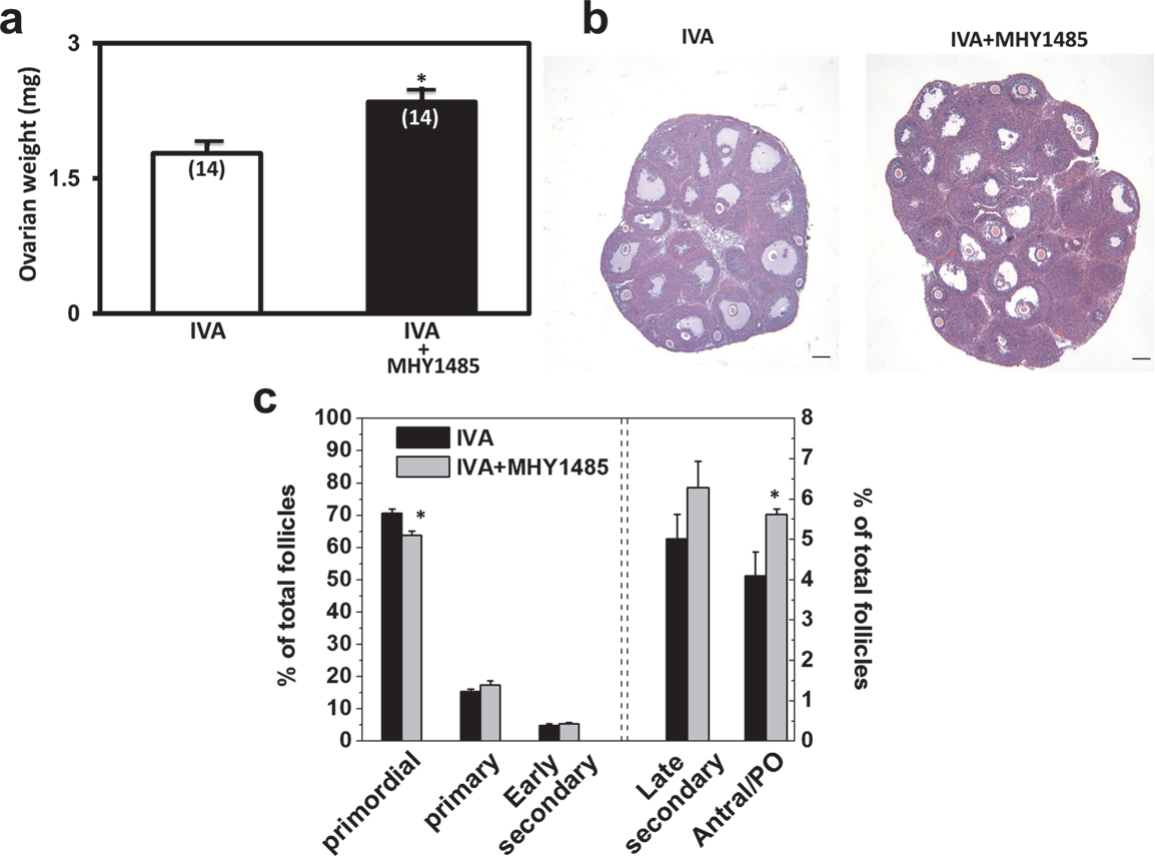
Additive effects of mTOR activation and AKT stimulation on follicle growth. A) Graft weight increases. Ovaries from day 10 mice were incubated with IVA drugs with or without MHY1485. Ovaries were then grafted into hosts treated daily with FSH for 5 days before determination of graft weights. * P<0.05. B) Ovarian histology; bars: 100 um. C) Follicle dynamics. PO: preovulatory. N = 5. * P<0.05.

Our earlier findings indicated the ability of AKT stimulators including PTEN inhibitors and phosphoinositol-3-kinase activators to promote secondary follicle growth [13],[16]. We, therefore, tested the combined effects of treating day 10 ovaries with both mTOR activator and AKT stimulators. Ovaries from day 10 mice were treated with optimal doses of the PTEN inhibitor bpv(hopic) and 740YP (an activator for phosphoinositol-3-kinase) routinely used in our in vitro activation (IVA) protocol [13] with or without MHY1485. As shown in Fig. 4A, co-treatment with MHY1485 and the IVA drugs further augmented graft weights as compared with those of grafts treated with IVA drugs alone. Histological analyses (Fig. 4B) and follicle counting (Fig. 4C) indicated increases in antral/preovulatory follicles, accompanied by a decrease of primordial follicles.

## Discussion

Our studies demonstrated the ability of an mTOR activator to stimulate the phosphorylation of mTOR and downstream proteins, to enhance secondary follicle growth in ovarian explant cultures, and to promote the generation of antral/preovulatory follicles in allografts. In addition to the PTEN-AKT-FOXO3 signaling pathway [8], suppression of mTORC1 activity by the TSC1–TSC2 complex in oocytes has been shown to be a prerequisite for maintaining the dormancy of primordial follicles based on extensive studies using mice with oocyte-specific deletion of TSC1 and TSC2 genes [6], [7]. Both PTEN and TSC1/2 suppress phosphorylation/activation of rpS6, but by regulating the phosphorylation of distinct threonine residues in S6K1 [7]. These earlier findings demonstrate the essential and coordinate roles of AKT and mTOR1 signaling pathways in the regulation of primordial follicle dormancy [17]. For secondary follicles, activation of either the mTOR [9] or the AKT [11] signaling pathway in granulosa cells of secondary follicles also promotes follicle development. Although both granulosa cells and oocytes were exposed to the present activation drugs, augmentation of follicle growth following treatment with mTOR and AKT signaling activators observed here likely reflect the stimulation of follicle growth mediated by granulosa cells due to the short duration of in vivo grafting.

Analyses of follicle dynamics demonstrated that short-term exposure to the mTOR activator promotes the growth of early secondary follicles to the antral/preovulatory stage in grafts. After stimulation of secondary follicles with MHY1485 to derive antral follicles, further treatment of animals with gonadotropins allowed the generation of multiple preovulatory follicles containing mature oocytes capable of developing into blastocysts and viable pups. Although the long-term health status and fertility potential of these pups remain to be evaluated, the present findings suggest that short-term exposure to mTOR signaling activators, similar to AKT signaling stimulators, could be the basis to develop future infertility therapies. In contrast to the ability of the mTOR activator to promote follicle growth described here, long-term injections with rapamycin (an inhibitor of mTOR signaling) lead to the suppression of follicle development in PTEN mutant mice [18] and prolong the fertile lifespan of aging rats by arresting follicle growth [19]. These findings underscore the important role of mTOR signaling in follicle growth. Although the present approach only involves short-term in vitro exposure of the mTOR activator, future studies on the long-term effects of MHY1485 and related compounds are needed before wide clinical application.

Although fertility is compromised in patients with primary ovarian insufficiency and middle-aged sub-fertile women, their ovaries still contain small number of preantral follicles [20]. Our earlier studies demonstrated that short-term exposure of human ovarian fragments with AKT stimulators (PTEN inhibitors and PI3K activators) promotes follicle growth and allow the generation of mature oocytes in ovarian grafts in a subpopulation of patients with primary ovarian insufficiency [16], leading to a new infertility treatment approach. The present observation further demonstrated the augmentation of follicle growth in ovarian grafts pre-incubated with both AKT stimulators and an mTOR activator. This transient and ovary-specific exposure to mTOR activators in vitro, when combined with treatment with AKT stimulators, could improve the success of infertility treatment as compared with the use of AKT stimulators alone [16]. Further studies on ovarian mTOR signaling could allow the development of new infertility strategies.

## Methods

### Animals and experiments

CD-1 and B6D2F1 mice were purchased from Charles River Laboratories (Wilmington, MA) and housed in animal facility of Stanford University under 12h light/dark with free access to water and food. Mice were treated in accordance with guidelines of the Stanford University Animal Research Committee. Stanford University Biosafety and Animal Research Committees approved all experimental protocols.

### Immunoblotting analysis

Ovaries from mice at day10 of age were treated with 10 μM MHY1485 (Millipore, Bedford, MA) for 3h and proteins were extracted using M-PER Mammalian Protein Extraction Reagent (Thermo, Rockford, IL) containing a protease inhibitor cocktail (Thermo). Protein concentrations in supernatants were determined by the bicinchoninic acid method (Pierce, Rockford, IL, USA). Equal amounts of protein lysates were loaded on 4–12% NuPAGE Bis-Tris gels (Invitrogen, Carlsbad, CA) in MOPS buffer and transferred to 0.45 μM pore nitrocellulose membranes (LI-COR, Lincoln, NE, USA). First antibodies were from Cell Signaling (Beverly, MA; #5536 for phosphor-mTOR; #2983 for mTOR; #9205 for phosphor-S6K1, #2708 for S6K1; #4858 for phospho-rpS6 (S235/6) and #2217 for rpS6) and IRdye 800 rabbit secondary antibody (926–32211) was from LI-COR. Images were generated using a LI-COR Odyssey infrared imager.

### Ovarian explant culture and follicle counting

Ovaries from day10 mice were placed on culture plate inserts (Millipore) and cultured in 400 μl of DMEM/F12 containing 0.1% BSA, 0.1% Albumax II, insulin-transferrin-selenium, 0.05mg/ml L-ascorbic acid and penicillin-streptomycin under a membrane insert to cover ovaries with a thin layer of medium. Ovaries were treated with 1–10 μM of MHY1485 and cultured for 4 days with medium change after 2 days of culture. Control ovaries were treated with solvent only. At the end of culture, ovaries were fixed with formalin before weighing. Some ovaries were paraffin-embedded and cut into continuous sections at 8 μm thickness. Sections were stained with hematoxylin and eosin for follicle counting, and in each section, only follicles with clearly stained oocyte nucleus were counted to avoid recounting of the same follicle.

### Ovarian tissue grafting

Paired ovaries from day10 mice were cultured on plate culture inserts in MEMα medium containing 3mg/ml BSA, 0.23mM sodium pyruvate, 50 μg/ml vitamin C, 30 mIU/ml FSH, 50 mg/L streptomycin sulfate and 75 mg/L penicillin G. Ovaries were treated with 3–20 μM MHY1485 for 48h with medium changes after 24h of culture. Paired ovaries (without or with MHY1485 treatment) from the same donor were grafted under kidney capsules of the same adult ovariectomized hosts (9–10 weeks of age) for 5 days with daily FSH injections (1 IU/animal). At the end of transplantation, grafts were collected for weight determination and histological analysis. To test the combined effects of mTOR activator and AKT stimulators, some ovaries from day 10 mice were treated with IVA drugs (PTEN inhibitor: (bpv(hopic) from Calbiochem at 30 μM for the first day and an activator for phosphoinositol-3-kinase740YP from Tocirs at 150 μg/mL for two days)[13], together with or without mTOR activator before grafting.

### In vitro fertilization and embryo transfer

Ovaries from B6D2F1 mice at 10 days of age were treated with 10 μM MHY1485 for 2 days, followed by transplantation into kidney capsules of hosts for 5 days. At day 5 after transplantation, animals were treated with 10 IU equine chorionic gonadotropin (eCG) for 48h, followed by an injection of 10 IU human chorionic gonadotropin (hCG). Twelve hour later, grafts were collected and oocytes were retrieved in the M2 medium (Cytospring, Mountain View, CA). As controls, B6D2F1 mice at day 25 of age were treated with 5IU eCG for 48h, followed by 5IU hCG before oocyte retrieval. For in vitro fertilization, sperm from B6D2F1 male mice were collected into human tubal fluid medium (Cytospring) and pre-incubated for 1h at 37C. Oocytes were then fertilized with sperm (2–3 X 10^5^/ml) for 6h, and inseminated oocytes were transferred into KSOM medium (Cytospring) for development into blastocysts. For embryo transfer, two-cell embryos were transferred into oviducts of pseudopregnant, 8-week-old CD1 nice pre-mated with vasectomized males of the same strain.

### Statistical analyses

Results are presented as the mean±SEM of three or more independent assays. Statistical significance was determined by using Student’s t test with P<0.05 being statistically significant.

## Supporting Information

S1 Fig Full-length immunoblotting figures for P-MTOR, MTOR, P-S6K1, S6K1, P-rpS6, and rpS6 are shown. (PDF)
