# Supplementary material for: Promotion of Ovarian Follicle Growth following mTOR Activation: Synergistic Effects of AKT Stimulators
Source: PLoS One. 2015 Feb 24;10(2):e0117769. doi: 10.1371/journal.pone.0117769 (PMC4340052; doi:10.1371/journal.pone.0117769)
Supplement: S1 Fig — (PDF) [file pone.0117769.s001.pdf]

Supplementary Materials:

Promotion of ovarian follicle growth following mTOR activation: synergistic effects of AKT stimulators

Yuan Cheng, Jaehong Kim, Xiao Xiao Li, Aaron J. Hsueh*

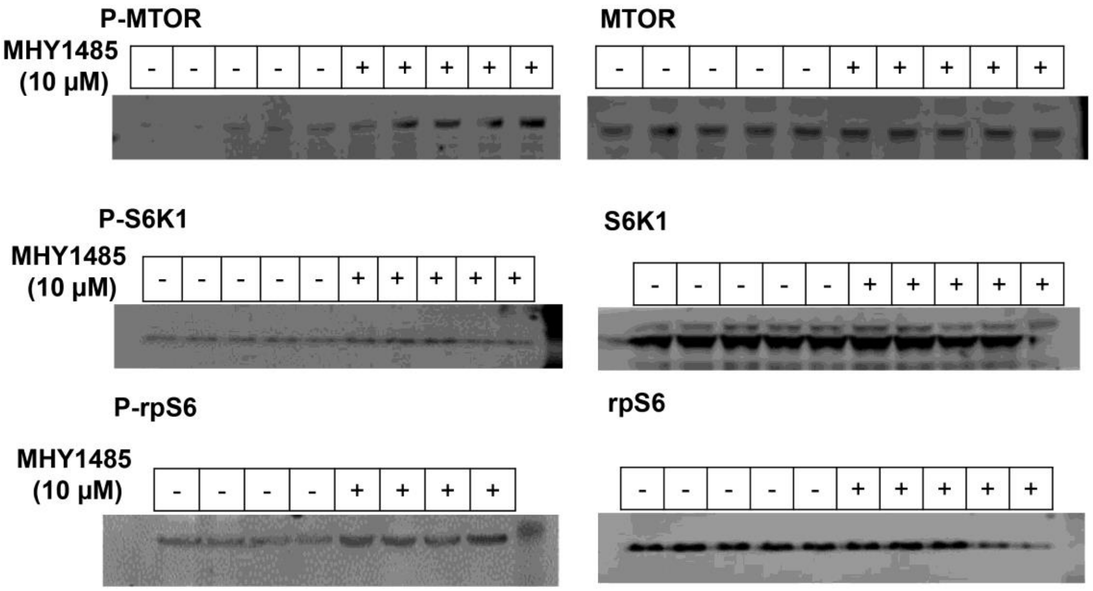

Supplementary Figure 1 Full-length immunoblotting figures for P-MTOR, MTOR, P-S6K1, S6K1, P-rpS6, and rpS6 are shown.
